# Effect of Melatonin and Epigallocatechin-3-Gallate Combination on In Vitro Maturation of Mouse Oocytes

**DOI:** 10.3390/ijms27021089

**Published:** 2026-01-22

**Authors:** Shuangshuang Li, Lili Chen, Yi Li, Lingyang Xu, Yan Chen, Yi Ma

**Affiliations:** 1 Tianjin Key Laboratory of Animal Molecular Breeding and Biotechnology, Tianjin Engineering Research Center of Animal Healthy Farming, Institute of Animal Science and Veterinary, Tianjin Academy of Agricultural Sciences, Tianjin 300381, China; lishuangshuang2024@163.com (S.L.); chenlili0609@163.com (L.C.); liyi202456@163.com (Y.L.); 2 Institute of Animal Sciences, Chinese Academy of Agricultural Sciences, Beijing 100193, China; xulingyang@163.com

**Keywords:** melatonin, epigallocatechin-3-gallate, oocyte maturation, oxidative stress

## Abstract

In vitro oocyte maturation (IVM) is a pivotal process influencing the success of embryo production in laboratory and clinical settings. However, oxidative stress (OS) often compromises oocyte quality during IVM. Antioxidants such as melatonin and epigallocatechin-3-gallate (EGCG) are known to mitigate OS by neutralizing reactive oxygen species (ROS) and bolstering antioxidant defenses. Despite extensive studies on their individual effects, the synergistic impact of melatonin and EGCG remains underexplored. Utilizing a mouse model, this study evaluated their combined effect on oocyte maturation, focusing on nuclear and cytoplasmic development, intracellular ROS, glutathione (GSH) levels, and subsequent embryonic competence. The results demonstrated that melatonin and EGCG significantly enhanced the polar body extrusion rate (*p* < 0.05), with the combination group achieving the highest rate of 91.96%. Cumulus expansion was observed to improve across all treated groups, with the combination treatment showing the highest cumulus expansion index (CEI) of 3.06. Furthermore, the combination treatment significantly reduced ROS levels and increased GSH content, indicating enhanced antioxidant capacity (*p* < 0.01). Embryonic development outcomes, including cleavage and blastocyst rates, were markedly higher in the combination group at 75.23% and 53.97%, respectively, demonstrating superior developmental potential (*p* < 0.01). These findings suggest that the melatonin–EGCG combination offers a novel and effective strategy to combat oxidative damage during IVM, thereby improving oocyte quality and embryonic development potential in mice.

## 1. Introduction

Oocyte maturation represents a fundamental biological prerequisite for fertilization and subsequent embryonic development [1]. In assisted reproductive technologies, particularly under in vitro maturation (IVM) conditions, the acquisition of oocyte developmental competence is frequently compromised by oxidative stress (OS), which results from an imbalance between the generation of reactive oxygen species (ROS) and the cellular antioxidant defense system [2]. Excessive ROS accumulation during IVM has been shown to induce oxidative damage to cellular macromolecules, disrupt mitochondrial integrity, and activate apoptotic signaling pathways, thereby impairing oocyte viability and developmental potential [3]. Multiple factors contribute to ROS overproduction in vitro, including metabolic activity of cumulus–oocyte complexes, culture medium components, and exposure to non-physiological environmental conditions. Although oocytes possess intrinsic antioxidant mechanisms, these defenses are often insufficient to maintain redox homeostasis during IVM, rendering OS a major constraint on IVM efficiency [4].

To counteract oxidative damage, antioxidant supplementation has been extensively investigated as a means to improve oocyte quality under in vitro conditions [5]. Among the antioxidants evaluated, melatonin has received particular attention due to its broad cytoprotective profile. Melatonin functions as an effective antioxidant through both direct and indirect mechanisms [6,7,8]. It directly neutralizes ROS and reactive nitrogen species (RNS) via electron donation, producing stable downstream products such as *N*^1^-acetyl-*N*^2^-formyl-5-methoxykynuramine (AFMK) [9]. In parallel, melatonin enhances endogenous antioxidant capacity by activating receptor-mediated signaling pathways, including the KEAP1–Nrf2 axis, leading to the upregulation of antioxidant enzymes such as HO-1 and NQO1 [10]. Moreover, melatonin contributes to mitochondrial homeostasis by promoting mitochondrial biogenesis and preserving membrane potential through the SIRT1/PGC-1α pathway, while inhibiting mitochondrial permeability transition pore (mPTP) opening [11,12]. Its anti-apoptotic effects are further supported by regulation of the Bcl-2/Bax ratio and inhibition of cytochrome c release [13], as well as attenuation of endoplasmic reticulum stress via the PERK-eIF2α-ATF4 pathway [14]. Consistent with these molecular actions, melatonin is present at relatively high concentrations in follicular fluid [15], and its receptors are found in granulosa and cumulus cells across species, including humans, pigs, cattle, and mice [16], supporting its physiological relevance in follicular development and oocyte protection [17]. Accordingly, melatonin supplementation during IVM has been reported to enhance oocyte maturation, improve intracellular glutathione levels, reduce oxidative damage, and promote subsequent embryo development [18,19].

Epigallocatechin-3-gallate (EGCG), the predominant catechin found in green tea (*Camellia sinensis*) [20,21], represents another antioxidant of interest in reproductive research. EGCG exerts antioxidative activity through several complementary mechanisms, including direct scavenging of free radicals, chelation of redox-active metal ions [22], and activation of the Keap1/Nrf2/ARE signaling pathway [23], thereby stimulating the expression of antioxidant enzymes such as superoxide dismutase, catalase, and glutathione peroxidase [24]. Within the context of IVM, experimental studies have demonstrated that EGCG supplementation reduces intracellular ROS levels, enhances mitochondrial membrane potential, and improves cleavage and blastocyst formation rates [25]. In addition to its antioxidant effects, EGCG exhibits a range of biological activities, including anti-microbial [26], anti-apoptotic [27], and anti-tumor properties [28].

Despite the documented benefits of melatonin and EGCG when applied individually, their combined use during IVM has not been systematically evaluated. Given their complementary biological properties, a potential synergistic effect between these two antioxidants is plausible. Moreover, previous evidence indicates that melatonin may alleviate adverse effects associated with high concentrations of EGCG while potentially enhancing its therapeutic efficacy [29]. Nevertheless, whether such a combinatorial antioxidant strategy can confer superior protection against oxidative stress during IVM remains unclear.

Therefore, the present study aims to investigate the combined effects of melatonin and EGCG on the IVM of mouse oocytes and their subsequent developmental potential. Nuclear maturation, cytoplasmic maturation, intracellular redox status, and early embryonic development were systematically evaluated. In addition, an NRF2 inhibitor (brusatol) was included to further explore whether the protective effects of melatonin and EGCG during IVM are mediated, at least in part, through the NRF2 signaling pathway [30]. Collectively, this study seeks to provide novel experimental evidence for the rational optimization of antioxidant-based IVM strategies, with potential implications for improving assisted reproductive technologies.

## 2. Results

### 2.1. Effect of Different Antioxidants on the PB1 Extrusion Rate in Mouse Oocytes

To investigate the effects of melatonin and EGCG combination on the IVM of mouse oocytes, the PB1 extrusion rates were evaluated in different groups (Table 1). Significant differences in the PB1 extrusion rates were observed between the control group and the melatonin, EGCG, and half-dose combination groups (*p* < 0.05). Among all groups, the half-dose combination group had the highest PB1 extrusion rate; however, this rate was not significantly different from those of the melatonin and EGCG groups (*p* > 0.05). In contrast, the PB1 extrusion rate in the brusatol group was significantly lower than that in the control group (*p* < 0.05).

### 2.2. Effect of Different Antioxidants on Cumulus Cell Expansion

**Figure 1:**
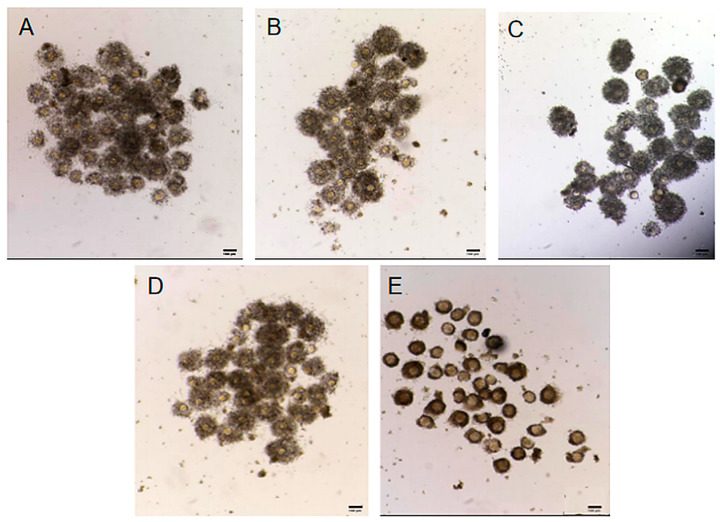
Cumulus cell expansion (50×) in the different groups. (**A**), control group; (**B**), melatonin group; (**C**), EGCG group; (**D**), half-dose combination group; (**E**), brusatol group (scale bar = 100 μm).

The effects of melatonin and EGCG on cumulus cell expansion were assessed based on the degree of expansion observed in each group (Figure 1, Table 2). The CEI was highest in the half-dose combination group; however, no significant differences were noted when compared with the CEIs of the control, melatonin, and EGCG groups (*p* > 0.05). In contrast, the CEI of the brusatol group was significantly lower than that in the control group (*p* < 0.05).

### 2.3. Effect of Different Antioxidants on GSH Levels in Oocytes

**Figure 2:**
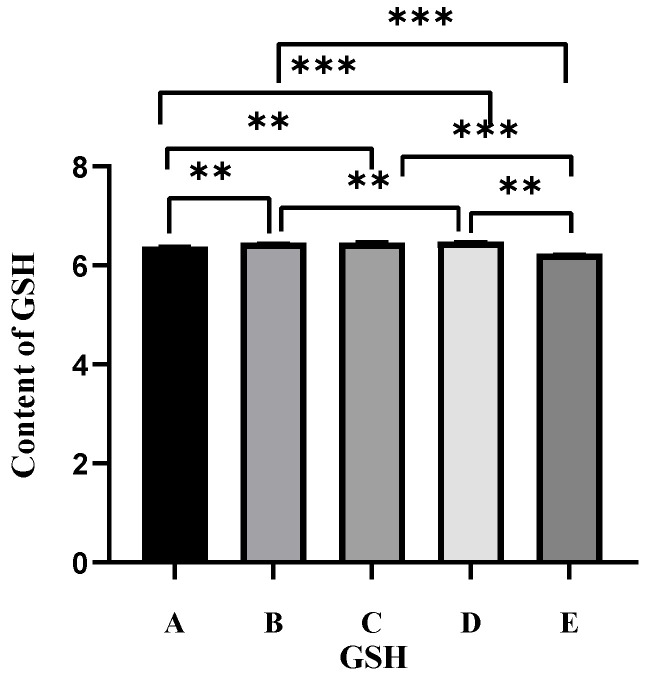
Effect of the different antioxidants on the GSH level in oocytes. A, control group; B, melatonin group; C, EGCG group; D, half-dose combination group; E, brusatol group. Note: ** *p* < 0.01, *** *p* < 0.001 indicate statistically significant differences between two groups.

By using GSH content as an indicator of antioxidant capacity, we analyzed whether the melatonin and EGCG combination affects GSH levels in oocytes. As shown in Figure 2, the half-dose combination group exhibited the highest GSH level, which was significantly higher than those observed in the control, melatonin, and brusatol groups (*p* < 0.01). No significant difference in GSH levels was observed between the half-dose combination and EGCG groups (*p* > 0.05).

### 2.4. Effect of Different Antioxidants on ROS Levels in Oocytes

**Figure 3:**
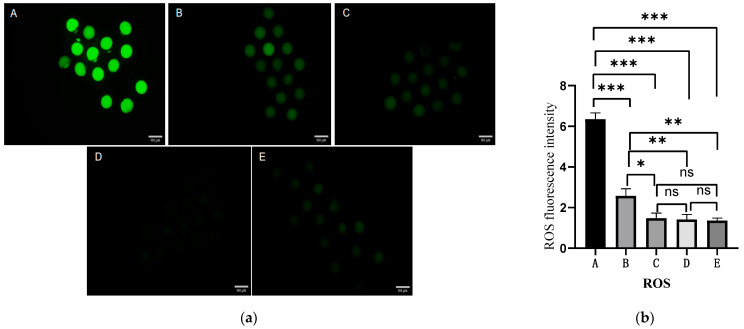
Effects of different antioxidants on reactive oxygen species (ROS) levels in oocytes. (**a**) Fluorescence images of ROS levels in oocytes from the five experimental groups (scale bar = 100 µm). (**b**) Quantitative analysis of ROS fluorescence intensity among experimental groups. A, control group; B, melatonin group; C, EGCG group; D, half-dose combination group; E, brusatol group. Note: * *p* < 0.05, ** *p* < 0.01, *** *p* < 0.001 indicate statistically significant differences between two groups; ns indicates no statistically significant difference.

Intracellular ROS levels were measured as an indicator of oxidative stress in oocytes from different groups. As shown in Figure 3, ROS levels in the half-dose combination group were significantly lower than that in the control and melatonin groups (*p* < 0.05). Although the EGCG group showed reduced ROS levels compared to the control group, the difference was not statistically significant (*p* > 0.05). The brusatol group exhibited the highest ROS levels among all groups.

### 2.5. Effect of Different Antioxidants on the Cleavage and Blastocyst Rates

As shown in Table 3 and Table 4, cleavage and blastocyst rates differed among the experimental groups. The half-dose combination group exhibited a significantly higher cleavage rate than the control, melatonin, EGCG, and brusatol groups (*p* < 0.05). Similarly, the blastocyst rate in the half-dose combination group was significantly higher than those in all other groups (*p* < 0.05).

## 3. Discussion

Oocyte quality is a critical determinant of successful fertilization and early embryonic development, and oxidative stress (OS) is widely recognized as a major factor compromising oocyte competence during in vitro maturation (IVM). Excessive accumulation of reactive oxygen species (ROS) under in vitro culture conditions can impair meiotic spindle organization, disrupt mitochondrial function, and interfere with cytoplasmic maturation, ultimately reducing fertilization efficiency and embryonic developmental potential [31,32]. Consequently, modulation of the intracellular redox environment has become a key strategy for optimizing IVM systems.

In the present study, combined supplementation with melatonin and epigallocatechin gallate (EGCG) resulted in coordinated improvements across multiple functional parameters, including nuclear maturation, intracellular redox balance, and early embryonic development. Rather than acting on a single endpoint, the combined treatment appeared to influence a network of interrelated processes essential for oocyte competence [29,33]. Melatonin is a well-established endogenous antioxidant that exerts both direct free-radical scavenging activity and indirect regulatory effects through intracellular signaling pathways and mitochondrial protection [34,35,36]. EGCG, a major polyphenolic catechin in green tea, exhibits strong antioxidant and metal-chelating properties and has been shown to reduce ROS accumulation and enhance glutathione (GSH) levels during oocyte maturation [24,37]. The concurrent improvement of ROS reduction and GSH elevation observed in this study suggests that the combination treatment promotes a more stable intracellular redox homeostasis than either compound alone.

The enhancement of first polar body (PB1) extrusion following combined antioxidant treatment indicates improved regulation of meiotic progression. Meiotic maturation is highly sensitive to oxidative imbalance, as ROS can induce spindle abnormalities and chromosome misalignment, leading to meiotic arrest or aneuploidy [38]. By simultaneously lowering ROS levels and increasing GSH availability, the melatonin–EGCG combination likely supports cytoskeletal integrity and redox-dependent enzymatic activities required for accurate meiotic division [39]. This integrated redox modulation may explain the tendency toward higher PB1 extrusion rates observed in the combination group.

Cumulus cells are somatic cells surrounding the oocytes and play crucial roles in the growth, meiotic maturation, ovulation, and fertilization of mammalian oocytes [40]. Although the increase in the cumulus expansion index (CEI) in the combination group did not reach statistical significance, the observed trend is consistent with improved cumulus–oocyte communication under optimized redox conditions. Notably, pharmacological inhibition of nuclear factor erythroid 2–related factor 2 (NRF2) signaling by brusatol markedly impaired cumulus expansion. NRF2 is a central transcriptional regulator of antioxidant defense genes, and its activity has been shown to be essential for maintaining redox balance in both oocytes and surrounding somatic cells [41,42]. The detrimental effects of brusatol observed in this study provide functional evidence that endogenous antioxidant signaling pathways contribute to cumulus cell function and oocyte maturation.

Importantly, the redox improvements achieved during IVM translated into enhanced embryonic developmental competence. Oocytes with balanced ROS–GSH homeostasis are more likely to support normal fertilization, cleavage, and blastocyst formation, as oxidative damage during oocyte maturation can have lasting effects on early embryogenesis prior to embryonic genome activation [43]. The significantly increased cleavage and blastocyst rates in the combination group indicate that optimizing antioxidant conditions during IVM can exert long-term benefits on embryo development. Similar improvements in embryonic outcomes have been reported with antioxidant supplementation, including melatonin and polyphenols, in various mammalian species [24,44].

These findings highlight the potential value of combination antioxidant strategies in refining IVM protocols. Compared with single-agent supplementation, combined approaches may achieve effective redox regulation at lower individual concentrations, thereby reducing the risk of pro-oxidant effects associated with excessive antioxidant exposure. Such strategies may be particularly relevant for assisted reproductive technologies (ART), where oocytes are inevitably exposed to non-physiological oxidative environments during in vitro handling and culture.

Nevertheless, certain limitations should be acknowledged. This study was conducted using a murine model, and species-specific differences in oocyte physiology may affect the direct translation of these findings to other mammals. In addition, although the involvement of redox regulation and NRF2 signaling is supported by the effects of brusatol, the precise molecular interactions underlying the synergistic action of melatonin and EGCG remain to be fully elucidated. Future studies integrating transcriptomic or proteomic analyses and exploring a broader range of antioxidant combinations may further clarify the regulatory networks involved and support the rational optimization of IVM systems.

## 4. Materials and Methods

### 4.1. Oocyte Collection

Kunming female mice, aged 6–8 weeks, were purchased from Weitonglihua Experimental Animal Technology Co., Ltd., Beijing, China. The mice were housed in a temperature-controlled room with a 12 h light/dark cycle and ad libitum access to food and water. To collect fully grown germinal vesicle (GV) oocytes, the mice were injected intraperitoneally with 10 IU of pregnant mare serum gonadotropin (Ningbo Hormone Product Company, Ningbo, China) to induce superovulation, which minimizes variability associated with the estrous cycle. After 46 h, the mice were euthanized by cervical dislocation, and the ovaries were harvested by opening the abdominal cavity. Antral follicles were punctured to collect cumulus–oocyte complexes (COCs).

### 4.2. IVM and IVF

For the IVM experiment, GV oocytes were washed at least three times with M2 medium droplets and immediately cultured in an oocyte maturation medium (8.5 mL M199 basal medium, 1.5 mL fetal bovine serum (FBS), 0.5 mL antibiotics (penicillin-streptomycin), and 1 ul growth factor (EGF)). Based on preliminary dose-optimization experiments, the oocytes were randomly divided into five groups: (1) control group, without the addition of melatonin or EGCG; (2) melatonin group that received 10^−6^ M melatonin alone; (3) EGCG group that received 20 μM EGCG alone; (4) half-dose combination group that received 5 × 10^−7^ M melatonin and 10 μM EGCG; and (5) brusatol group that received 100 nM of the NRF2 inhibitor brusatol. Oocyte maturation rates were assessed after 15 h of treatment.

For the IVF experiment, Healthy male mice with a proven mating history were selected and then euthanized. The abdominal cavity was opened, and the epididymis and tail epididymis were removed and dissected under a microscope. Sperms were incubated in a sperm energy-harvesting fluid for 1 h. IVF droplets (Nanjing Aibei Biology Co., Ltd., Nanjing, China) were prepared for the control, melatonin, EGCG, half-dose combination, and brusatol groups, either 4 h prior to or overnight. Capacitated sperms from each group were mixed with mature oocytes and placed in the IVF droplets for 4.5 h. Subsequently, the fertilized eggs were transferred to in vitro compartmentalization droplets. The fertilized eggs developed to the 2-cell stage after 24 h of incubation and reached the blastocyst stage after 96 h of incubation.

### 4.3. Determination of the PB1 Extrusion Rate

To assess the effect of the melatonin and EGCG combination on the nuclear maturation of oocytes, the PB1 extrusion rate was determined. After 15 h of culture, cumulus cells surrounding the mature COCs were gently removed by repeated pipetting. The denuded oocytes were then examined under a microscope (Nikon, Tokyo, Japan) to count the number of oocytes with PB1 extrusion.

### 4.4. Cumulus Cell Expansion

Cumulus cell expansion was evaluated after 15 h of IVM using the following scoring criteria:

grade 0: no change in cumulus cells; grade 1: expansion of cumulus cells by 1–2 layers; grade 2: 50% expansion of cumulus cells; grade 3: expansion of all cumulus cells, except those of the corona radiata; and grade 4: complete expansion of all cumulus cells [45]. The cumulus expansion index (CEI) was calculated as follows:

CEI = (0 × number of COCs with grade 0) + (1 × number of COCs with grade 1) + (2 × number of COCs with grade 2) + (3 × number of COCs with grade 3) + (4 × number of COCs with grade 4) [46].

### 4.5. Detection of ROS Levels

To investigate the effect of melatonin and EGCG combination on ROS levels in in vitro matured oocytes, cumulus cells were removed by gentle pipetting. The denuded oocytes were washed three times with PBS containing 0.1% polyvinyl alcohol (PVA). The oocytes were then incubated with 10 μM 2′,7′-dichlorofluorescein diacetate for 20 min at 37 °C in the dark. Subsequently, the oocytes were washed three times with PBS containing 0.1% PVA. Finally, the oocytes were observed under a fluorescence microscope (Nikon). The fluorescence intensities of the oocytes from the melatonin, EGCG, half-dose combination, and brusatol groups were analyzed using ImageJ software (version 1.49, National Institutes of Health, Bethesda, MD, USA) and normalized to that of the control group oocytes.

### 4.6. Estimation of GSH Levels

To estimate GSH levels, cumulus cells surrounding mature oocytes were removed using hyaluronidase. Oocytes with PB1 were washed three times with PBS and collected in a centrifuge tube using a collecting needle. GSH level estimation was performed on the same day when possible; otherwise, the samples were stored at −80 °C for no more than 10 days. The washed oocytes were centrifuged, and the supernatant was collected. A protein extraction removal reagent (M solution) was added to the supernatant, and the mixture was vortexed thoroughly. The samples were then subjected to two rapid freeze–thaw cycles by using liquid nitrogen and a 37 °C water bath. Subsequently, the samples were centrifuged at 10,000× *g* for 10 min at 4 °C. The obtained supernatant was used for total GSH determination.

A standard curve was constructed using 2, 5, 10, 15, and 25 µM concentrations of GSH solutions in accordance with the instructions provided in the GSH Assay Kit (Beyotime Biotechnology, Shanghai, China). The samples or standards were added to a 96-well plate and mixed. Next, 150 µL of total GSH detection working solution was added and mixed, and the plate was incubated for 5 min at room temperature. Next, 50 µL of 0.5 mg/mL NADPH solution was added and mixed. Following incubation for 25 min at room temperature, the absorbance was measured at 412 nm by using a microplate reader.

### 4.7. Statistical Analysis

Each experiment was repeated at least three times with newly collected oocytes from different batches of mice, representing biological replicates. Data were presented as mean ± standard deviation and were tested for normality. Data were statistically analyzed using one-way ANOVA or independent sample *t*-test with SPSS 22.0 software, with *p* < 0.05 considered statistically significant.

## 5. Conclusions

The combination of melatonin and epigallocatechin-3-gallate (EGCG) effectively enhances the in vitro maturation of mouse oocytes by reducing oxidative stress, improving nuclear and cytoplasmic maturation, and increasing developmental potential. These findings suggest that this antioxidant combination could serve as a valuable approach to improve oocyte quality and embryo development in assisted reproductive technologies.

## Figures and Tables

**Table 1:** First polar body extrusion rates of the different groups.

Group	Number of Oocytes	PB1 Extrusion Rate
Control group	126	66.66 ± 0.51 b
Melatonin group	128	89.79 ± 0.58 a
EGCG group	134	89.56 ± 0.40 a
Half-dose combination group	125	91.96 ± 0.57 a
Brusatol group	129	22.43 ± 0.98 c

Note: Different letters (a, b, c) in the same column represent significant differences (*p* < 0.05).

**Table 2:** Cumulus expansion index of the different groups.

Group	Cumulus Expansion Index
Control group	3.02 ± 0.03 a
Melatonin group	3.03 ± 0.05 a
EGCG group	3.02 ± 0.04 a
Half-dose combination group	3.06 ± 0.05 a
Brusatol group	1.87 ± 0.05 b

Note: Different letters (a, b) in the same column represent significant differences (*p* < 0.05).

**Table 3:** The cleavage rates of the different groups.

Group	Number of Embryos	Cleavage Rate
Control group	102	56.82 ± 0.44 b
Melatonin group	95	65.31 ± 0.51 a
EGCG group	102	63.74 ± 0.31 a
Half-dose combination group	97	75.23 ± 0.39 a
Brusatol group	99	17.12 ± 1.00 c

Note: Different letters (a, b, c) in the same column represent significant differences (*p* < 0.05).

**Table 4:** The blastocyst rates of the different groups.

Group	Number of Embryos	Blastocyst Rate
Control group	72	30.94 ± 0.28 b
Melatonin group	74	46.88 ± 0.42 b
EGCG group	76	44.85 ± 0.23 b
Half-dose combination group	75	53.97 ± 0.47 a
Brusatol group	70	4.45 ± 0.33 c

Note: Different letters (a, b, c) in the same column represent significant differences (*p* < 0.05).

## Data Availability

The original contributions presented in this study are included in the article. Further inquiries can be directed to the corresponding authors.
